# Bridging the Digital Divide: Community-Engaged Strategies for Implementing Technology in Rural Adult Day Centers

**DOI:** 10.1093/geroni/igaf122.989

**Published:** 2025-12-31

**Authors:** Tina Sadarangani

**Affiliations:** New York University, New York, New York, United States

## Abstract

Successfully embedding technology into long-term care settings requires intentional, evidence-based community engagement strategies that address the unique needs of providers, caregivers, and persons living with dementia. This presentation will explore the implementation of CareMobi, a HIPAA-compliant mobile health application designed to enhance communication between adult day centers and family caregivers, within a rural, under-resourced adult day center setting and identify key components of a community-engaged approach to technology implementation that prioritized collaborative design, staff training, and iterative adaptation to fit the center’s workflows. Drawing from qualitative and quantitative data, we identify successful approaches to recruitment, intervention design, and implementation of CareMobi that address digital literacy among staff and caregivers, infrastructure limitations, and the importance of trust-building in rural communities. We identified strategies that supported sustained engagement, including co-developing implementation protocols and recruitment strategies with frontline staff, an iterative user centered process for designing the app, leveraging existing caregiving networks, and aligning technology use with reimbursement structures and regulatory requirements. Findings from 2 centers and n = 17 participants, suggest that CareMobi improved information-sharing between adult day center staff and families, enabling earlier identification of health changes and care coordination needs. By providing insights into real-world application, this presentation will contribute to the broader discussion of how researchers and practitioners can effectively introduce and sustain technology in community-based long-term care settings. The lessons learned from this rural implementation will offer a framework for scalable, equitable adoption of digital health tools across diverse adult day center environments.

